# Viral engagement with host receptors blocked by a novel class of tryptophan dendrimers that targets the 5-fold-axis of the enterovirus-A71 capsid

**DOI:** 10.1371/journal.ppat.1007760

**Published:** 2019-05-09

**Authors:** Liang Sun, Hyunwook Lee, Hendrik Jan Thibaut, Kristina Lanko, Eva Rivero-Buceta, Carol Bator, Belen Martinez-Gualda, Kai Dallmeier, Leen Delang, Pieter Leyssen, Federico Gago, Ana San-Félix, Susan Hafenstein, Carmen Mirabelli, Johan Neyts

**Affiliations:** 1 KU Leuven–University of Leuven, Department of Microbiology and Immunology, Rega Institute for Medical Research, Laboratory of Virology and Chemotherapy, Leuven, Belgium; 2 Department of Biochemistry and Molecular Biology, Huck Institutes of the Life Sciences, The Pennsylvania State University, University Park, Pennsylvania, United States of America; 3 Instituto de Química Médica, Consejo Superior de Investigaciones Científicas (IQM, CSIC), Madrid, Spain; 4 Department of Biomedical Sciences, School of Medicine and Health Sciences, University of Alcalá, Unidad Asociada IQM-CSIC, Madrid, Spain; 5 Department of Medicine, The Pennsylvania State University College of Medicine, Hershey, Pennsylvania, United States of America; University of California at Los Angeles, UNITED STATES

## Abstract

Enterovirus A71 (EV-A71) is a non-polio neurotropic enterovirus with pandemic potential. There are no antiviral agents approved to prevent or treat EV-A71 infections. We here report on the molecular mechanism by which a novel class of tryptophan dendrimers inhibits (at low nanomolar to high picomolar concentration) EV-A71 replication *in vitro*. A lead compound in the series (MADAL385) prevents binding and internalization of the virus but does not, unlike classical capsid binders, stabilize the particle. By means of resistance selection, reverse genetics and cryo-EM, we map the binding region of MADAL385 to the 5-fold vertex of the viral capsid and demonstrate that a single molecule binds to each vertex. By interacting with this region, MADAL385 prevents the interaction of the virus with its cellular receptors PSGL1 and heparan sulfate, thereby blocking the attachment of EV-A71 to the host cells.

## Author summary

Enterovirus A71 (EV-A71) is the virus responsible for most of the severe forms of hand, foot and mouth disease (HFMD) associated with neurological involvement and mortality in young children under the age of 5. Seasonal outbreaks of HFMD -with a 2–3 years epidemic cycle- are recurring around the world, especially in the Asia-Pacific region. To date, no antiviral agent has been approved for the treatment of EV-A71 infections. Here, we report on a recently uncovered class of tryptophan dendrimers with an extraordinary antiviral activity *in vitro* against circulating EV-A71 clinical isolates. Mode of action studies revealed that this class of compounds targets the 5-fold vertex of EV-A71, in turn blocking receptor binding. Our finding may open an entirely novel line of research and largely aid in anti-enterovirus drug development.

## Introduction

Since the first large outbreak in 1997, enterovirus A71 (EV-A71) (genus *Enterovirus*, family *Picornaviridae*) has been reported to cause 2–3 year cyclic epidemics in the Asia-Pacific region [1,2]. In the last two decades, the increasing number of EV-A71 cases and the spread of the virus across Asia have raised major concerns about its pandemic potential. The virus is primarily transmitted by the oral-fecal route [3,4]. Most EV-A71 infections are characterized by mild symptoms, with the typical signs of the hand, foot and mouth disease (HFMD): slight fever, red rashes on the palms of hand and soles of feet, and ulcers in the mouth. However, EV-A71 infections are also associated to severe neurological complications (such as encephalitis, aseptic meningitis and poliomyelitis-like syndrome) and acute pulmonary edema, which may be highly limiting and fatal particularly in children under the age of 5 years [5,6]. In 2010, a large outbreak of HFMD in China resulted in an estimated 1.7 million cases and 905 deaths [7] and an outbreak in Cambodia in 2012 resulted in the death of 54 children [8,9]. A sub-genogroup C4 EV-A71-inactivated vaccine has recently been approved in China, but worldwide coverage and long-term protection still need to be addressed [10–12]. There are no antiviral agents approved against EV-A71 nor against any other enteroviruses.

EV-A71 has been reported to bind to several cell surface receptors, including scavenger receptor B2 (SCARB2) [13,14], P-selectin glycoprotein ligand-1 (PSGL1) [14,15] and heparan sulfate (HS) glycosaminoglycan [16]. Other host factors such as cyclophilin A, annexin II, sialylated glycans, vimentin, nucleolin, fibronectin and prohibitin have also been reported to promote infection, although their importance in viral entry is still less noted [17–23]. It has been shown that EV-A71 interaction with PSGL1 on leukocytes requires the presence of sulfated tyrosine (Tyr) residues at the N-terminal region of PSGL1 [24] and depends on two highly conserved lysine residues, VP1_244K and VP1_242K, near the 5-fold vertex of the viral capsid [25]. A spatially close residue, VP1_145, is another determinant for PSGL1 binding [26]. Similarly to PSGL1, HS has also been proposed to interact near the 5-fold vertex of the viral capsid [15,16,22].

A well-known class of inhibitors of entero- and rhinovirus entry (such as pirodavir, pleconaril and vapendavir) bind into a hydrophobic pocket under the floor of the viral “canyon” formed by VP1. Drug binding prevents receptor interaction and/or increases particle stability, which in turn blocks the conformational changes required for viral uncoating [27–32].Despite their notable potency *in vitro*, none of these compounds reached advanced clinical trials.

Attachment of EV-A71 to host cells can also be blocked by suramin and other sulfated and sulfonated analogs, including NF449, which bind the positively charged residues clustered at the five-fold axis of the viral capsid, in turn preventing PSGL1 and HS attachment [33–35]. According to the proposed mechanism of action by Ren *et al*., the VP1_145 residue was found to be critical for the inhibitory profile of suramin [33]. On the other hand, amino acid changes at position VP1_244 and VP1_98 modulated the antiviral effect of NF449 [34]. These findings reveal a pivotal role of the 5-fold vertex of the viral capsid for binding to host receptors and lodging molecules able to inhibit EV-A71 replication.

Recently, we discovered a class of inhibitors with dual activity against HIV and EV-A71 [36,37]. The lead compound of this family, MADAL385, is a tetrapodal derivative with a pentaerythritol core, 4 trivalent spacer arms and 12 tryptophan (Trp) residues on the periphery [38]. Because the tryptophan dendrimers are linked to the central scaffold through their amino groups, their carboxylates are free and exposed to the solvent. Our earlier biological studies demonstrated that MADAL derivatives inhibit HIV entry into its target cell by interaction with glycoproteins gp120 and gp41 of the viral surface [36]. For EV-A71, we demonstrated that MADAL derivatives exhibit low micromolar activity against the lab-adapted strain BrCr and low-nanomolar/high-picomolar activity against a large panel of EV-A71 clinical isolates from different genogroups and various geographic origins [38]. Structure-activity relationship (SAR) studies on the periphery and central scaffold of MADAL highlighted the importance of free carboxylic groups for optimal antiviral activity, those carried by Trp or Tyr residues.

In the present work, we elaborate on the precise molecular mechanism of action of the lead compound MADAL385 by means of *in vitro* biological assays, cryo-EM analysis and molecular modeling. Our data support a model of activity by which a single MADAL385 molecule binds on each of the 5-fold vertices of the EV-A71 capsid, thereby blocking the engagement of the virus with host receptors PSGL1 and/or HS.

## Results

### MADAL385 efficiently blocks EV-A71 attachment and internalization

**Fig 1:**
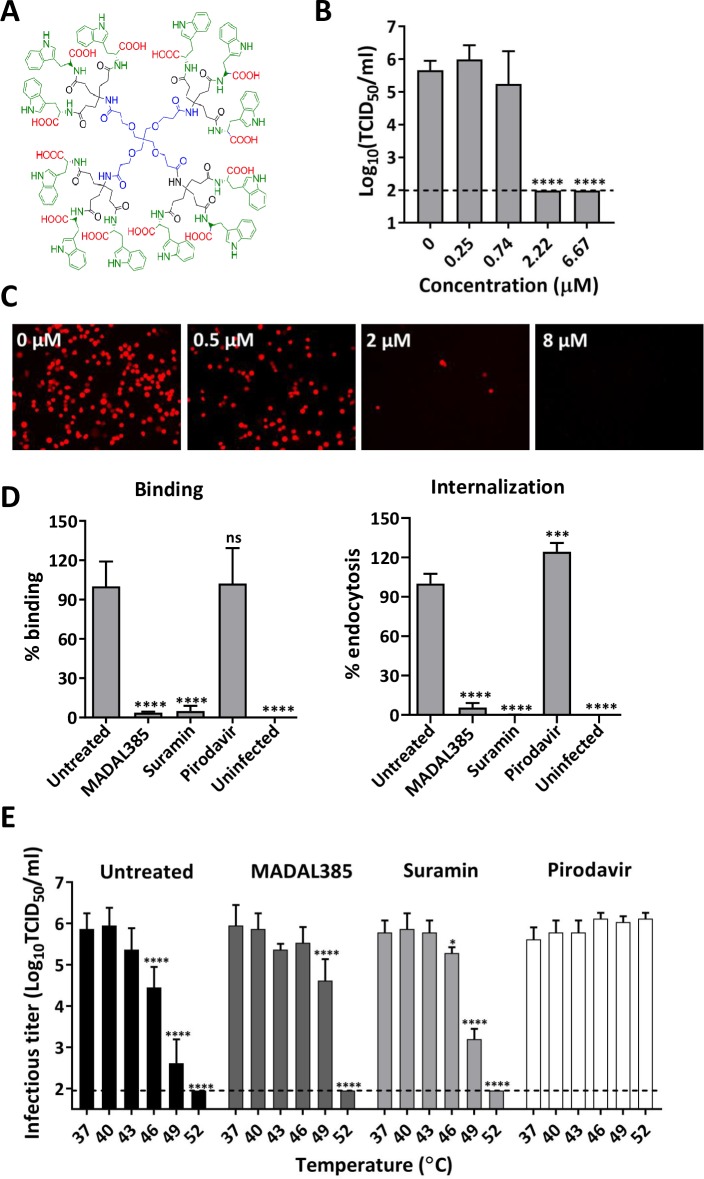
Characterization of the *in vitro* anti-EV-A71 activity of MADAL385. **(A)** Structural formula of MADAL385. **(B)** Concentration dependent inhibition of EV-A71 BrCr replication. RD cells were infected with EV-A71 BrCr (MOI = 0.1) for 1h in presence of the compound, after which the inoculum was removed, and cells were treated with serial dilutions of MADAL385. After 3 days, viral progeny was quantified by end-point titration. Statistical analysis was performed using the one-way ANOVA test; ****p<0.0001. **(C)** Antiviral activity of MADAL385 in the context of mCherry-EV-A71 reporter virus infection (MOI = 0.1). Images were taken with a fluorescence microscope at 24h post infection. **(D)** Binding (left panel) and internalization (right panel) inhibition assays. The graph represents the RNA levels relative to the untreated-infected group (VC). Statistical analysis was performed using the one-way ANOVA test; ***p<0.001, ****p<0.0001. **(E)** Thermo-stability assay. EV-A71 viruses, in the presence/absence of compounds (at a concentration of 50-fold *in vitro* EC_50_: 15 μM MADAL385, 150 μM suramin and 15 μM pirodavir), were subjected to heat-inactivation at the indicated temperature for 2 min, after which the samples were cooled down on ice. Viral titers were quantified by end-point titration. Pirodavir and suramin were used as reference compounds. Statistical analysis was performed using the two-way ANOVA test in reference to the Pirodavir treament; *p<0.05, ****p<0.0001. Error bars represent the mean ± standard deviation (SD) of at least 2 independent experiments with three replicates.

From the series of Trp dendrimers endowed with high *in vitro* potency against EV-A71 replication, MADAL385 (**Fig 1A**) was selected for further mechanistic studies (EC_50_ against the lab-adapted BrCr strain: 0.29 ± 0.07 μM, CC_50_: 30.0 ± 2.5 μM) [38]. In a cytopathic effect (CPE)-based reduction assay, MADAL385 is ~1,800 to ~20,000-fold more effective against a representative EV-A71 clinical isolate (EV-A71_11316, geno-group B2) than two earlier reported EV-A71 inhibitors, namely pirodavir [31] and suramin [39] (**S1 Fig**). MADAL385 inhibits, in a dose-dependent manner, (i) the formation of infectious EV-A71 BrCr particles (**Fig 1B**) and (ii) the replication of a mCherry-reporter EV-A71 BrCr (**Fig 1C**). MADAL385 inhibits the early stages of EV-A71 replication [38] and to precisely determine which step of viral entry is targeted, binding and internalization assays were performed with pirodavir and suramin as reference. MADAL385 (10 μM) markedly reduces -akin to suramin- the binding of EV-A71 to the host cells and prevents as well the internalization of the virus (**Fig 1D**). In contrast, pirodavir does not show any inhibitory activity in either the binding or the internalization assays, which is in line with the reported mechanism of action (i.e. inhibition of viral uncoating). By tightly binding in the hydrophobic pocket, “classical’ capsid binders such as pirodavir and pleconaril increase the rigidity of the viral particle and, as a consequence, its resistance to heat inactivation. MADAL385, in marked contrast to the capsid-binder pirodavir protects the virus only slightly against heat inactivation (**Fig 1E**). Taken together, these results suggest that MADAL385 blocks viral attachment by binding to the virus particle in a region outside the hydrophobic pocket.

### MADAL385-resistant and -sensitive EV-A71 strains carry mutations in the gene encoding the viral protein VP1

**Fig 2:**
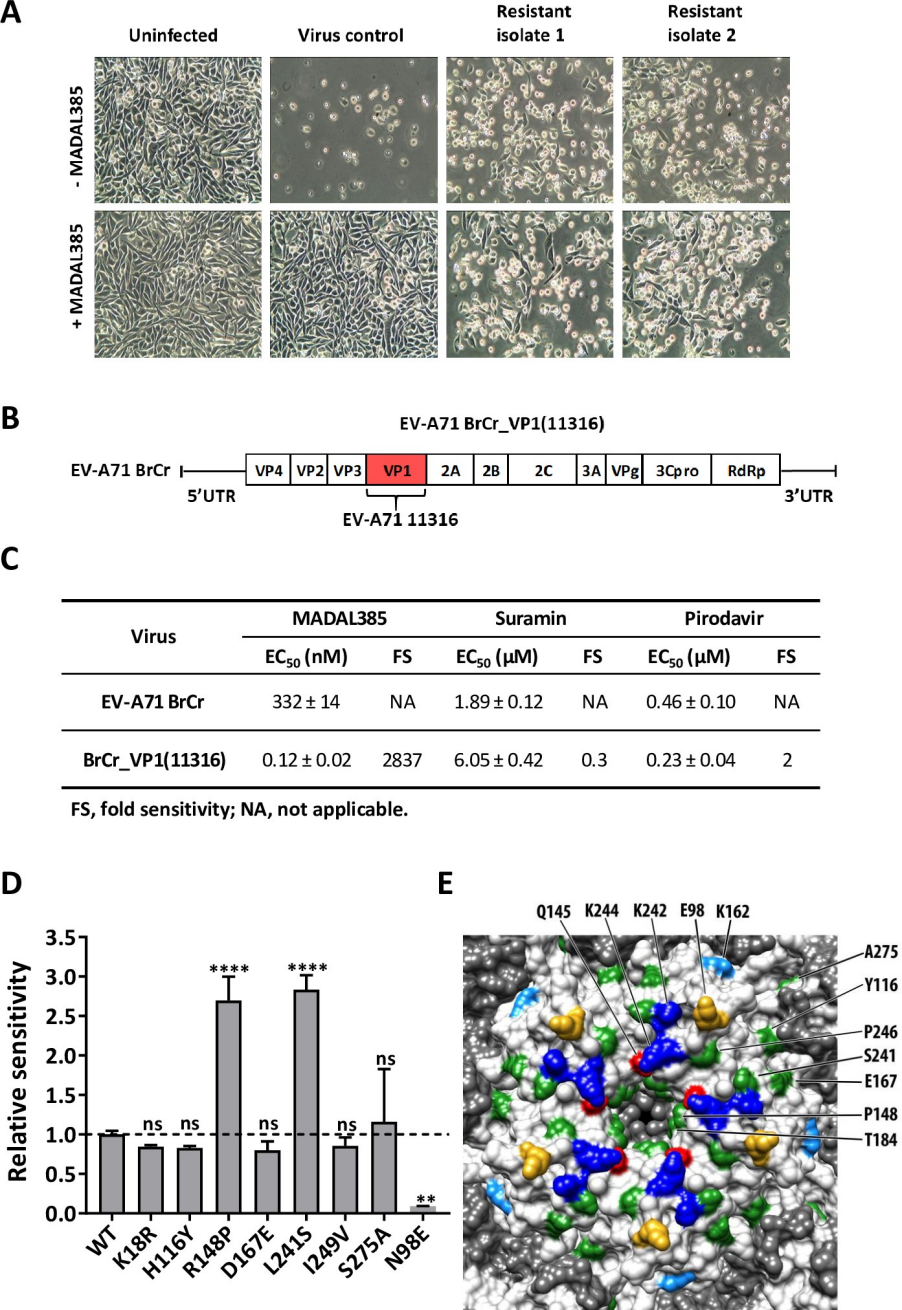
Selection and characterization of MADAL385-resistant and -sensitive EV-A71 strains. (A) EV-A71 BrCr and resistant strains were used to infect RD cells in presence or absence of MADAL385 (3 μM). Images were taken at 1-day post infection. (B) Schematic representative of the recombinant EV-A71 BrCr_VP1(11316). (C) *In vitro* EC_50_s of MADAL385, suramin and pirodavir against EV-A71 BrCr and EV-A71 BrCr_VP1(11316) infection in RD cells. (D) Relative sensitivity of 8 EV-A71 single mutants to MADAL385. Data were normalized on the EC_50_ of EV-A71 BrCr (WT). Statistical analysis was performed using the one-way ANOVA test relative to the wild-type; **p<0.01; ****p<0.0001; ns, not significant. (E) Surface rendering of the EV-A71 pentamer was generated using USCF Chimera (PDB ID: 3VBS). VP1 residues that bind PSGL1 and HS (K242 and K244, blue) or determine the receptor-binding phenotype (Q145, red) or identified as HS-binding (K162, light blue), or complementing HS binding (E98, gold) are colored accordingly and indicated (top). Positions associated with MADAL385 resistant/sensitive phenotype are colored in green and indicated (right). Amino acids were labeled according to the corresponding residues of EV-A71_11316. Error bars represent the mean ± SD of at least 2 independent experiments with 3 independent replicates each.

To gain first insights into the putative binding site of MADAL385, two independent, MADAL385-resistant strains were generated via a stepwise clonal resistance selection procedure (**Fig 2A**). Full genome sequence analysis revealed two amino acid replacements in the capsid protein VP1 of the BrCr strain: S184T and P246S (**Table 1**). To confirm that S184T and P246S are responsible for resistance to MADAL385, the single or double mutant(s) were engineered by site-directed mutagenesis in the EV-A71 BrCr strain infectious clone. All mutant variants show replication kinetics comparable to that of the wild-type virus (**S2A Fig**). The susceptibility of the single S184T and P246S mutants to MADAL385 is decreased by 7- and 17-fold, respectively; the double mutant (S184T_P246S) confers the highest (32-fold) resistance to the compound (**Table 2)**. Next, to address the role of viral VP1 in antiviral susceptibility, we engineered a recombinant virus by swapping the VP1 of the BrCr strain with that of the EV-A71 11316 clinical isolate, against which MADAL385 is ≥1000-fold more potent (BrCr strain EC_50_: 0.28 ± 0.01μM versus 11316 strain EC_50_: 0.21 ± 0.03nM) (**Fig 2B**). The susceptibility of the recombinant EV-A71 BrCr_VP1 (11316) strain to MADAL385 increases dramatically to the level observed with the clinical isolate (EC_50_: 0.12 ± 0.02nM) (**Fig 2C**). A comparison of sequences of BrCr and selected circulating EV-A71 clinical isolates (including EV-A71_11316) revealed 8 amino acid differences in the viral VP1 (**S2B Fig**). These residues were individually engineered into the BrCr infectious clone. Only R148P and L241S mutants show an increased sensitivity to the drug, without however fully restoring the susceptibility of the EV-A71_11316 strain (**Fig 2D**). Altogether, these data further point towards the VP1 as the molecular target of MADAL385. Notably, most of the sensitivity and resistance residues are located in the proximity of the 5-fold vertex of the EV-A71 capsid, in a region known to be involved in HS and PSGL1 receptor binding [25,40] (**Fig 2E**). As expected, both the MADAL385-resistant strain and the more susceptible recombinant strain EV-A71 BrCr_VP1(11316) were shown to retain wild-type sensitivity to the capsid binders pirodavir and vapendavir. However, to our surprise, no cross-resistance with suramin was observed, despite the fact that this molecule was earlier reported to interact with the positively charged region surrounding the 5-fold axis of the capsid [33]. In addition, when combined, MADAL385 and suramin result in a strong synergistic *in vitro* antiviral effect with mean volume of 771 μM^2^% (**S2C Fig**). According to MacSynergy method [41], values over 100 μM^2^% indicate strong and biologically relevant synergy. The lack of cross-resistance and the *in vitro* synergistic effect thus suggest a different mode of antiviral action of MADAL385 and suramin.

**Table 1:** Two amino acid (AA) substitutions in VP1 were identified in 2 MADAL385-resistant EV-A71 strains.

Pool	Protein	AA Substitution	Nucleotide	
			Substitution	Position
1	VP1	S184T	TCC -> ACC	2988
		P246S	CCC -> TCC	3174
2	VP1	S184T	TCC -> ACC	2988
		P246S	CCC -> TCC	3174

The whole genomes of EV71 BrCr Wild-type (WT) and compound-resistant strains were sequenced.

**Table 2:** Susceptibility of reverse-engineered EV-A71 strains to MADAL385 and other reference compounds.

Virus	MADAL385		Suramin		Pirodavir		Vapendavir		Rupintrivir	
	EC_50_ (μM)	FR	EC_50_ (μM)	FR	EC_50_ (μM)	FR	EC_50_ (μM)	FR	EC_50_ (nM)	FR
										
EV-A71 BrCr	0.28 ± 0.01	NA	4.62 ± 0.49	NA	0.33 ± 0.06	NA	1.91 ± 0.16	NA	2.51 ±0.05	NA
										
VP1_S184T	1.99 ± 0.10	7.1	4.53 ± 0.22	1.0	0.28 ± 0.06	0.8	1.93 ± 0.17	1.0	2.47 ±0.09	1.0
										
VP1_P246S	4.71 ± 0.18	16.8	3.28 ± 0.37	0.7	0.24 ± 0.10	0.7	1.47 ± 0.55	0.7	3.41 ±0.32	1.4
										
VP1_S184T_P246S	8.93 ± 0.53	31.9	3.76 ± 0.15	0.8	0.41 ± 0.10	1.2	2.12 ± 0.32	1.1	2.93 ± 0.12	1.2

Averages and SD (standard deviation) were calculated from data obtained from at least two independent antiviral assays. FR, fold resistance; NA, not applicable.

### Cryo-EM study reveals binding of MADAL385 at the 5-fold axis of the viral capsid

**Fig 3:**
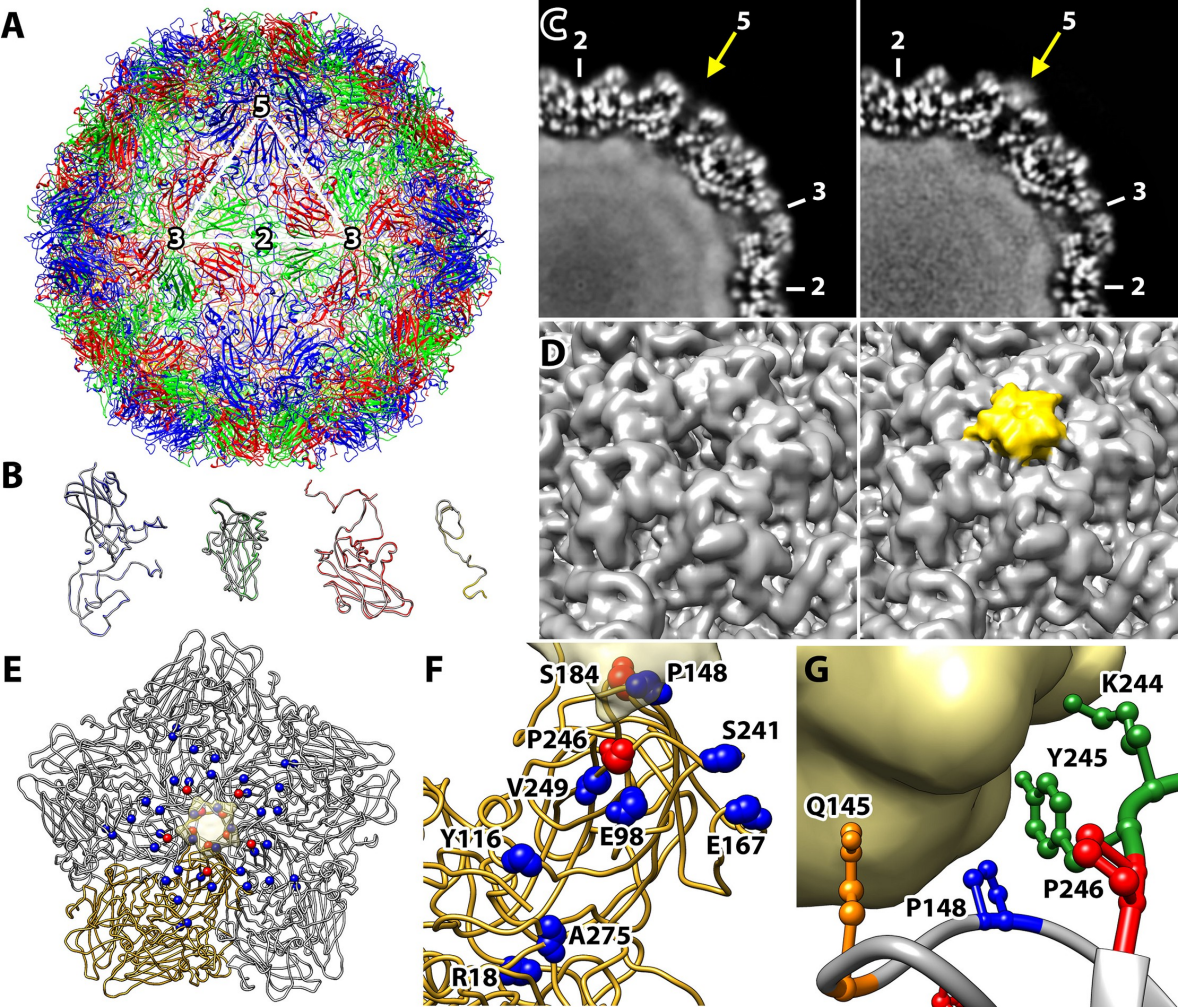
The atomic resolution structures were solved for EV-A71_11316 strain and MADAL385 to interpret the interaction of virus and compound. (A) The ribbon diagram (VP1-4 as blue, green, red, and yellow, respectively) of the atomic model of the EV-A71_11316 clinical isolate capsid built into the cryo-EM 3D reconstruction map. Asymmetric unit and symmetry axes are indicated. (B) The four capsid proteins, VP-1 to VP-4 (from left to right), from the EV-A71_11316 strain and the EV-A71-MADAL385 complex were super-imposed yielding an average RMSD 0.292 Å. The central sections of the cryo-EM densities (C) and the surface rendered maps (D) for EV-A71 alone (left) and EV-A71-MADAL385 complex (right) show that the complex includes strong extra densities on the capsid surface at the 5-fold icosahedral symmetry axis (yellow arrow) (symmetry axes indicated). The surface rendered maps displayed in orientation indicated in (A) clearly distinguishes the extra densities at the 5-fold symmetry axes (yellow). (E and F) The residues identified from the resistant (red spheres) and susceptible (blue spheres) mutants were mapped on the VP-1 pentamer (E) and monomer (F). The monomer in (F) represents the monomer colored in gold in (E). The segmented drug density was shown in a transparent tan surface rendering. (G) Residues of one VP1 molecule comprising the 5-fold illustrates P148 (blue), K244 and Y245 (green) which were identified in the continuous density envelope connected to the MADAL385 densities at a contour level of σ = 1. The location of Q145 (orange) is also shown.

To define the binding area of MADAL385 and to understand the possible mechanism underlying resistance and susceptibility to the compound, cryo-EM single particle analysis of MADAL385 bound to the EV-A71_11316 strain was performed. Purified EV-A71_11316 particles were vitrified before and after incubation with MADAL385 (cryo-EM 2D class average images **S3 Fig**). The corresponding three-dimensional (3D) maps were reconstructed at 3.3 and 3.6 Å resolutions, respectively, by applying icosahedral-symmetry averaging and using the images from a Falcon-3 direct electron detector **(S1 Table)**. The atomic models of free and drug-bound EV-A71_11316 (**Fig 3A**) superimpose with an overall RMSD value of 0.292 Å **(Fig 3B)**, indicating that the incubation with MADAL did not cause any significant conformational change on the viral capsid proteins. In the two atomic models, the pocket factor lipid (sphingosine) has slightly different conformations, but the corresponding cryo-EM densities have similar intensities (**S4 Fig**), suggesting that the drug binding likely induces subtle conformational changes around the pocket region but does not initiate pocket factor release. When the two 3D reconstructions were compared, strong extra densities were identified on the 5-fold vertices of the EV-A71_11316-MADAL385 complex (**Fig 3C**), indicating that MADAL385 binds on the 5-fold vertex. The drug density fills the pore on the 5-fold symmetry axis and is connected to the capsid density. Also, the density intensity is comparable to that of the capsid shell (**Fig 3D**). Thus, MADAL385 binds on the 5-fold vertex and full saturation of the binding sites is achieved by the incubation. In particular, the intensity of MADAL385 density was highest at the symmetry axis, suggesting that the bound drug molecule occupies the very centric area of the 5-fold vertex. Due to steric hindrance and electrostatic repulsion, these observations suggest that only one molecule of MADAL385 binds on each vertex (**Fig 3D**).

### Cryo-EM analysis and molecular dynamics characterization of EV-A71-MADAL385 interaction

The location of the two MADAL385-resistance mutations and the eight sensitivity mutations were mapped on the atomic model of EV-A71 (**Figs 2E and 3E**). Among those, the sensitivity variant VP1_148P, is located on the surface of the channel at the 5-fold axis (**Figs 2E and 3F**) and is found within the electron density envelope connected to MADAL385 density. The presence of a positively charged arginine residue in the BrCr strain (instead of a proline) may provide an explanation for the reduced drug sensitivity of this lab strain. Two other residues, VP1_244K and 245Y, with their corresponding side-chains extended towards MADAL385, are also found within the density envelope at the interface between capsid and drug (**Fig 3G**). The two resistance mutations, VP1_184T and 246S, are located closely to the drug density but are not found within the density envelope **(Fig 3F and 3G)**, therefore interactions between MADAL385 and those residues cannot be verified or excluded based on the cryo-EM result. The rest of the other sensitivity mutations are distal from the cryo-EM densities corresponding to MADAL385, suggesting origins for the susceptible phenotypes (such as adaptive mutations) other than the physical binding of the drug. Further interpretation was limited because of the icosahedral symmetry averaging applied during the cryo-EM reconstruction on the asymmetrically bound drug molecules.

To complement the cryo-EM analysis, molecular dynamics (MD) simulations in explicit water were performed on both the free MADAL385 (for conformational sampling) and its complex with the VP1 pentamer (for assessing pose stability and characterization of intermolecular interactions). In the case of the Cα-restrained complex, the pentaerythritol core of the tetrapodal MADAL385 was initially lodged in the external pore region corresponding to the highest electron density while three of its ‘legs’ projected into the inter-subunit crevices and the remaining one occupied the outer part of the pore (**S5 Fig**). In this location at the 5-fold axis, the Trp carboxylates and indole rings of MADAL385 establish interactions with the positively charged residues and the hydrophobic cavities. This binding mode is reminiscent of the binding to sulfated Tyr residues **(S1 Movie)** of PSGL1 and HS. To identify the VP1 residues at the 5-fold axis that contribute predominantly to MADAL385 binding, van der Waals and solvent-screened electrostatic interactions, together with the cost of desolvation upon complex formation, were calculated [42]. In line with the cryo-EM analysis, VP1_244K, 245Y, 145Q, and 148P are the residues with which MADAL385 establish the strongest favorable interactions (**S6 Fig**).

### MADAL385 blocks EV-A71 attachment to HS and PSGL1 receptors

**Fig 4:**
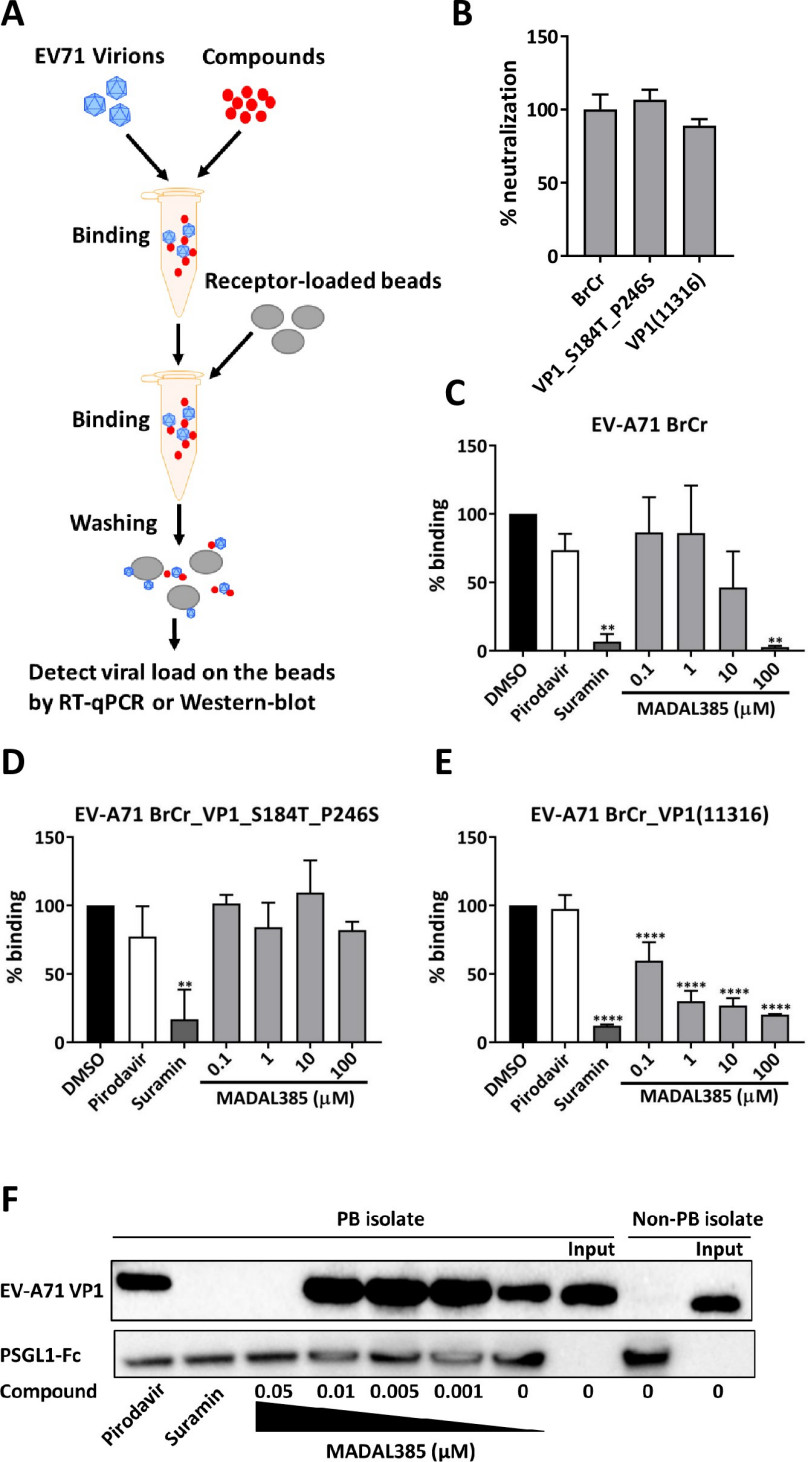
MADAL385 blocks the engagement of EV-A71 to its receptors. (A) Scheme of the receptor binding inhibition assay setup. (B) Relative neutralization of EV-A71 BrCr, VP1_S184T_P246S and VP1(11316) to heparin. Data are expressed in percentage compared to EV-A71 BrCr (WT). Heparin-coated beads were incubated with (C) EV-A71 BrCr, (D) EV-A71 VP1_S184T_P246S and (E) EV-A71 BrCr_VP1(11316) in presence of indicated concentration of MADAL385 and other reference compounds, binding was measured by RT-qPCR. The graph represents the RNA binding levels relative to the untreated group (DMSO). Error bars represent the mean ± SD of at least 2 independent experiments with at least 2 replicates. Statistical analysis was performed using the one-way ANOVA test; **p<0.01, ****p<0.0001. (F) Lysate of HEK293 cells overexpressing PSGL1-Fc was incubated with protein G beads in TBS buffer overnight at 4°C. EV-A71 BrCr (non-PB) and EV-A71_11316 (PB) were added in the presence of selected compounds. Attachment was determined by Western-blot with EV-A71 VP1-specific antibody or anti-human IgG Fc antibody.

**Fig 5:**
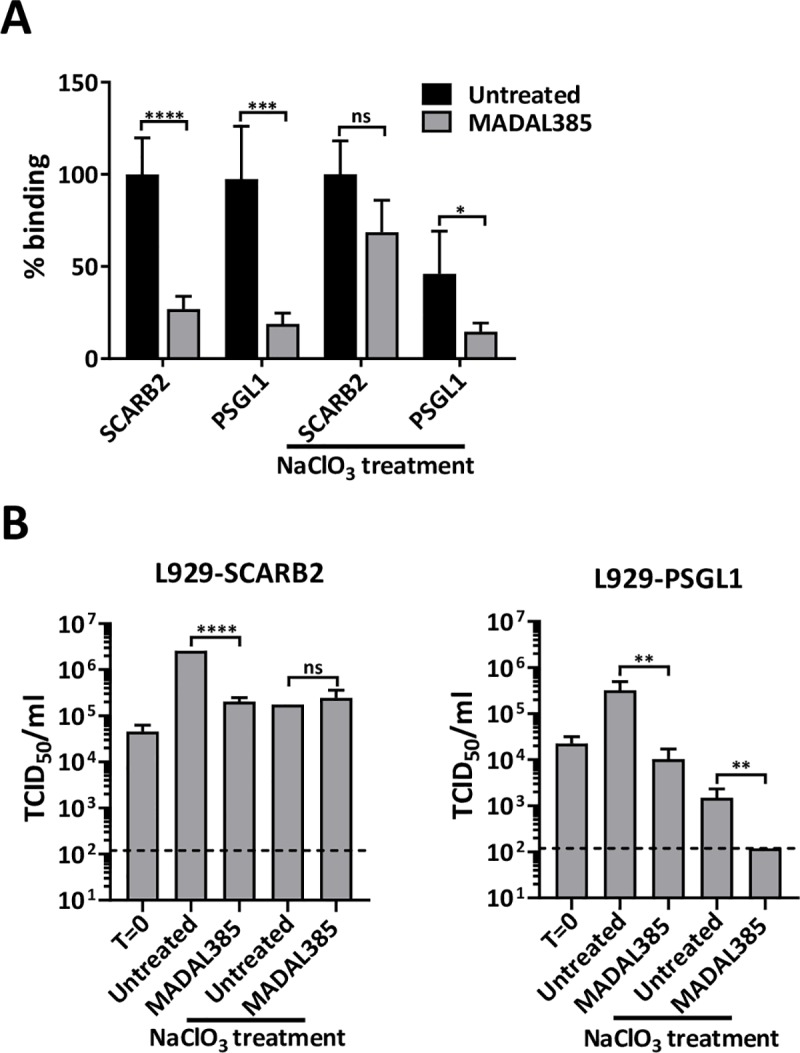
Anti-EV-A71 effect of MADAL385 on L929-SCARB2 and PSGL1 cells. (A) Binding assay of EV-A71_812 to L929-SCARB2 and PSGL1 overexpressing cells in either the presence or absence of MADAL385. A condition with cells pre-treated with sodium chlorate (NaClO3, 50mM) which prevents cell-surface sulfation was also included. The graph represents the RNA levels of bound virus relative to the L929-SCARB2 infected and L929-SCARB2 infected pre-treated with NaClO3 condition, respectively. (B) MADAL385 inhibits EV-A71 infection of L929-SCARB2 and PSGL1 cells. L929-SCARB2 and PSGL1 cells were infected with EV-A71_812 in presence or absence of MADAL385 and in presence or absence of NaClO3 (50mM). Twenty-four hours (24h) after infection, viral progeny was quantified by end-point titration. Error bars represent the mean ± SD of at least 2 independent experiments with 3 replicates each. Statistical analysis was performed using the Student t- test; ns, not significant; *p<0.05; **p<0.01; ***p<0.001; ****p<0.0001.

HS and PSGL1 have been proposed to play critical roles in the early steps of EV-A71 infection by interacting with the positive charges at the 5-fold vertex of the EV-A71 capsid. In addition, our structural analysis suggests that the binding site of MADAL385 overlaps with the binding sites of both receptors. We hence hypothesized that MADAL385 exerts its antiviral activity by blocking virus attachment to PSGL1 and/or HS. The experimental setup is depicted in **Fig 4A**. Binding-inhibition assays were performed with heparin or PSGL1-coupled beads in either the presence of MADAL385 or reference compounds and the extent of inhibition was quantified by RT-qPCR or Western-blot. First, the ability to bind soluble heparin was assessed by neutralization assay for the BrCr, MADAL385-resistant and sensitive EV-A71 strains. Similar sensitivity is noted across the different viruses (**Fig 4B**). Next, HS-binding inhibition assay revealed that binding affinity between heparin and EV-A71 BrCr is gradually lost with increasing concentration of MADAL385 (**Fig 4C**). In contrast, MADAL385 does not inhibit heparin binding of the resistant strain (EV-A71 VP1_S184T_P245S), even at the highest concentration tested (**Fig 4D**). Interestingly, and in line with the high susceptibility to the antiviral action of MADAL385, binding of the recombinant EV-A71 BrCr_VP1(11316) strain to heparin is inhibited by MADAL385 more efficiently as compared to the BrCr strain, with more than 50% binding affinity lost at low micromolar concentrations (**Fig 4E**). As expected, the pocket binder pirodavir does not significantly affect binding of EV-A71 to heparin, whereas suramin completely blocks heparin binding of all viruses (**Fig 4C–4E**). Next, the effect of MADAL385 on the interaction of PSGL1 with EV-A71 was studied. For this purpose, PSGL1-Fc was over-expressed in HEK293T cells (**S7 Fig**) and concentrated on protein G beads. MADAL385, akin to suramin, blocks binding of the EVA71-PB strain to PSGL1 in a concentration-dependent manner. The capsid binder pirodavir has, as expected, no effect on this binding event (**Fig 4F**). To determine whether MADAL385 affects attachment to PSGL1 and HS during infection, we employed human SCARB2- or PSGL1-overexpressing L929 cells. EV-A71_812 strain, a PSGL1 binding strain sensitive to MADAL385 (EC_50_: 1.78 ± 0.81nM) was used to study binding to the cell receptors in presence of MADAL385 (0.1 μM). To account for (and exclude) the contribution of HS, L929-SCARB2 and PSGL1 cells were treated in parallel with sodium chlorate (NaClO_3_, 50mM), an inhibitor of cell-surface sulfation. MADAL385 greatly reduced the binding of EV-A71 to both L929-SCARB2 and PSGL1 cells; however, in NaClO_3_-treated cells, MADAL385 effectively reduced binding of EV-A71 to L929- PSGL1 but not to L929-SCARB2 cells (**Fig 5A**). Quantification of the infectious content of EV-A71_812 in L929-SCARB2 and PSGL1 cells treated with MADAL385 in presence of NaClO_3_ recapitulate the data of the binding assay; i.e. MADAL385 is only effective against EV-A71 in L929- PSGL1 cells (**Fig 5B**). These data support the hypothesis that MADAL385 blocks EV-A71 infection by preventing viral attachment to either HS, PSGL1 or both.

## Discussion

We report here on the mechanism of action of MADAL385, the lead compound of a novel class of tryptophan dendrimers with exquisitely potent *in vitro* antiviral activity against EV-A71. Cryo-EM studies revealed that the highly conserved lysine residue at position 244 of VP1 (VP1_244K), near the icosahedral 5-fold vertex, is closely connected to the density of MADAL385. This residue also plays a key role in the interaction of EV-A71 with PSGL1 and HS. Both receptors are sulfated molecules (*i*.*e*. endowed with a negative charge at physiological pH) whose interaction with the positively charged VP1_244K capsid residue is thought to involve a strong electrostatic interaction. As a result of this high-affinity interaction, we showed that MADAL385 inhibits EV-A71 binding with PSGL1 and HS. Together with biochemical evidence, we also demonstrate that MADAL385 inhibits the binding of EV-A71 to human SCARB2- or PSGL1-expressing L929 cells. We observed that the activity of MADAL385 in L929-SCARB2 cells was exclusively dependent on the inhibition of HS binding since the activity of MADAL385 was lost in cells treated with sodium chlorate (NaClO_3_), a molecule that prevents cell-surface sulfation. In addition, this experiment also demonstrates the importance of HS binding for efficient entry and replication of EV-A71 in both L929-SCARB2 and L929-PSGL1 overexpressing cells.

Recent SAR studies performed with MADAL derivatives [37] point to the crucial role of the carboxylic acid groups for the antiviral efficacy. The importance of these carboxylates is corroborated by the lack of activity observed with the corresponding tryptamine (a “decarboxylated” analogue of Trp) and methyl ester derivatives (COOCH_3_ instead of COOH) [37]. The nature of the amino acid side chains is also very important for activity since the indole ring of Trp is preferred, most likely due to its relative polarity and hydrogen-bonding potential, particularly towards the hydroxyl of Thr141 (according to our MD simulations). These observations suggest that the carboxylic acid (-COOH) or carboxylate (-COO^-^) groups of MADAL385 can mimic the sulfate groups (-SO_3_H or -SO_3_^-^) of human PSGL1 or HS. By competing with the sulfate groups, MADAL385 may prevent virus attachment to these host receptor(s) and thereby the entry into and infection of host cell.

In agreement with the cryo-EM results showing that MADAL385 is lodged in the external pore region, the MD simulations revealed the preference of the drug for binding inside the cavity lined by the adjoined ^141^TPTGQVVP^148^ and ^242^QSKYP^246^ loops of the five VP1 subunits. Three of the MADAL385 ‘legs’ projected into the inter-subunit crevices and the remaining one occupied the outer part of the pore. It seems, therefore, that a certain conformation of these two exposed VP1 loops is necessary for MADAL385 binding, most likely for providing relative accessibility of VP1_244K to interact with the negative charges of MADAL385. We propose that the location of the MADAL385-sensitive variants VP1_148P and VP1_245Y is critical for stacking and establishing van der Waals interactions with the indole moieties of MADAL385. Furthermore, our data demonstrate that the MADAL385-resistant variants may only reduce viral sensitivity to MADAL385 in the context of the BrCr strain VP1. Indeed, the clinical isolate EV-A71_11316 carries the VP1_184S residue, a resistant variant for the BrCr strain.

Suramin and its derivative NF449 are known to specifically interact with residues VP1_145Q and VP1_98E_244K at the 5-fold vertex, respectively [33,35]. Of interest, we show that the susceptibility to suramin is not affected in the presence of both S184T and P246S amino acid substitutions. In addition, we observed a prominent *in vitro* antiviral synergistic effect between MADAL385 and suramin. These results indicate that, despite the common binding site for these two classes of drugs, the subtleties of their binding modes are different, which is not entirely surprising given their very distinct chemical structure. However, based on molecular modelling considerations, the simultaneous binding of MADAL385 and suramin on the same 5-fold vertex seems most unlikely due to the large size and negatively charged character of both entities.

Cyclophilin A is a newly reported uncoating regulator for EV-A71 entry, and its binding site is very close to that of PSGL1 and HS on EV-A71 virion [22]. The MADAL class of tryptophan dendrimers may thus have the potential to block Cyclophilin A binding. However, the Cyclophilin A inhibitor cyclosporin A (CsA) did not affect binding of EV-A71 BrCr to RD cells (**S8 Fig**) nor could we observe any antiviral activity of CsA or Debio-025 (another specific Cyclophilin A inhibitor) against BrCr and the clinical isolates 11316 and 812 (CsA EC_50_>30μM and Debio-025 EC_50_>21μM), suggesting that this host factor does not play a prominent role in the entry of the strains that we used in our study.

Medicinal chemistry efforts are currently ongoing to simplify and reduce the backbone of MADAL385 without affecting the antiviral activity. Half-sized compounds (~1500 Da versus 3575.84 Da) have now been identified that are equipotent to MADAL385 against EV-A71 and that have little or no adverse effect on the host cells (at concentrations up to 100 μM). *In vivo* studies to assess tolerability and antiviral efficacy will be complementary to medicinal chemistry efforts to pursue the development of this class of compounds as novel EV-A71 antiviral agents.

## Materials and methods

### Cells and viruses

RD cells (Human rhabdomyosarcoma cells) and Hela cells, obtained from ATCC, were cultured in DMEM (Life Technologies) supplemented with 10% heated-inactivated fetal bovine serum (FBS); Enterovirus A71 BrCr strain (EV-A71 BrCr), a kind gift from Prof. F. van Kuppeveld (University of Utrecht), was propagated on RD cells with 2% FBS-DMEM. L929-human SCARB2 cells and L929-human PSGL1 cells were kindly provided by Prof. Satoshi Koike (Tokyo Metropolitan Institute of Medical Science, Japan) and were grown in DMEM supplemented with 10% FBS, 1% sodium bicarbonate, 1% L-glutamine and 10μg/ml puromycin. To reduce sulfate from the cell surface, 50mM of sodium chlorate (NaClO_3_) was added to the culture medium one week before the experiment. EV-A71 clinical isolates were obtained from Dr. Shih-Cheng Chang (Chang Guang University) or purchased from The National Collection of Pathogenic Viruses (NCPV).

### Compounds, plasmids and reagents

The Trp-containing dendrimer MADAL385 was synthesized as described previously [38]. Vapendavir and pirodavir were kindly provided by Aviafen Therapeutics and A. Muigg, respectively. Suramin sodium salt and heparin sodium were purchased from Sigma-Aldrich. All compounds were dissolved in DMSO and stored at 4°C. The plasmid pEV-A71 (Nagoya-VP231) was generously provided by Dr. Arita (National Institute of Infectious Disease, Japan) and was modified to carry the viral genome of EV-A71 BrCr (pEV-A71-BrCr) by classic cloning. For the construction of EV-A71BrCr-mCherry, the genome of EV-A71 BrCr was inserted to a pShuttle-BAC vector. In the spacer region between the 5'UTR and VP4, mCherry gene was inserted and flanked by 2A^pro^ cutting sites. The plasmids pLX304-PSGL-1 was purchased from DNASU, and vector pFUSE-hIgG1-Fc1 was from InvivoGen. PSGL-1 extracellular region was cloned into the expression vector pFUSE-hIgG1-Fc1 to create recombinant protein with human IgG Fc-tag at the C-terminal domain (pPSGL1-hFc). The EV-A71 VP1 purified MaxPab rabbit polyclonal antibody was obtained from Abnova. Goat anti-Human IgG Fc cross-absorbed secondary antibody, HRP was purchased from Thermo Scientific. Heparin Sepharose CL-6B and Dynabeads Protein G were supplied by Pharmacia and Thermo Scientific, respectively.

### Multi-cycle cytopathogenic effect (CPE) reduction assay

CPE reduction assays were performed as described previously [43]. Briefly, RD cells in 96-well cell culture plates were seeded, after which serials of diluted compounds and EV-A71 inoculum were added. CPE was quantified by MTS assay at 3 days post infection and expressed as percentage of untreated controls. The 50% effective concentration (EC_50_) was calculated by logarithmic interpolation and is defined as the concentration at which the virus-induced CPE is reduced by 50%.

### EV-A71 quantitative RT-PCR (qRT-PCR)

EV-A71 viral RNA was isolated using NucleoSpin RNA kit (Macherey-Nagel) according to the manufacturer’s protocol. The following primers and probe were used for EV-A71 BrCr qRT-PCR: EV-A71 forward primer 5’ CCGCATTGAACCACTGTAATTT 3’, reverse primer 5’ GGAGCCAACGTGATAGTGATAG 3’ and probe 56-FAM/ACTATTGGT/ZEN/GGTGCCTATCA/3IABKFQ

### *In vitro* pull-down assay

Recombinant plasmid pPSGL-1-Fc was transfected in HEK-293T cells. After 36-48h, cells were harvested and lysed (1% Triton X-100 in NTE buffer supplemented with protease inhibitor cocktail) for 30 min in ice. Insoluble cell debris was removed by centrifugation 15,000 × g for 15 minutes. To confirm pPSGL-1 expression, the supernatant was subjected to Western-blot with goat anti-Human IgG Fc antibody. After confirmation, the soluble recombinant PSGL1-Fc was incubated with protein G Dynal beads (Sigma) overnight at 4° C on a rotary mixer. Unbound PSGL1-Fc was removed after 3 washes with cold TBS. The EV-A71 viruses were mixed with indicated concentrations of compounds at 37° C for 1 hour. Next, the beads loaded with PSGL1 were incubated with viruses in presence/absence of compounds at 4° C for 2 hours. After 3 washing with TBS to remove the unbound virus, beads were treated with 2x laemmli buffer (Sigma-Aldrich) and subject to 10% SDS-PAGE and further Western-blot with EV-A71_VP1 MaxPab rabbit polyclonal antibody and goat anti-Human IgG Fc antibody. For heparin-Sepharose coated beads, after 3 washing with TBS to remove the unbound virus, lysis buffer was added and viral RNA was extracted and quantified by real time qRT-PCR.

### Virus purification

EV-A71_11316 was purified as described previously [44]. Briefly, EV-A71_11316 was propagated in HeLa cells for 24 h. The media and cells were collected and processed by freezing and thawing three times. Cell debris was pelleted by centrifugation and the supernatant was precipitated with sodium chloride and polyethylene glycol (PEG) 8000. After ultracentrifugation through a 30% sucrose buffer cushion, the pellets were resuspended and applied to a 10 to 35% K-tartrate step gradient. The virus was collected and dialyzed against 10 mM Tris, 200 mM NaCl, 50 mM MgCl_2_, pH 7.5.

### Electron microscopy data collection

EM samples were prepared and data sets were recorded at the Pennsylvania State University—Huck Institutes of the Life Sciences in the following way: prior to incubation and vitrification of the sample, the virus buffer was exchanged to phosphate-buffered saline (PBS). MADAL385 was incubated at 2.8 μM with about 58 nM of the virus at 37°C for 1 hour, which equates to four copies of molecule per each of vertex on the virus capsid. Three microliters of each sample was pipetted onto a Quantifoil R2/1 grid (Quantifoil Micro Tools GmbH, Jena, Germany), blotted to remove excess, and plunge-frozen in liquid ethane using a Vitrobot (Thermo Fisher, USA). Grids were imaged in a Titan Krios G3 under automated control of the FEI EPU software. An atlas image was assembled from micrographs taken at 165x magnification on a FEI BM-Ceta camera, and suitable areas were selected for imaging on the FEI Falcon 3EC direct electron detector. The microscope was operated at 300 kV with a 70 μm condenser aperture and a 100 μm objective aperture. Magnification was set at 59,000x yielding a calibrated pixel size of 1.1 Å. Images were recorded in movie mode saving 44 fraction images, each fraction including 56 frames. The total accumulated exposure was 46 e^-^/Å^2^.

### Map and structure accession numbers

The cryo-EM density maps for the EV-A71 virus and the two virus-complexes are available at the Electron Microscopy Data Bank via accession codes EMD-7905 (sharpened and unsharpened virus maps) and EMD-7913 (sharpened and unsharpened virus-MADAL385 complex maps). The atomic coordinates for the viruses built in the two maps are available at the PDB via accession codes 6DIJ (virus) and 6DIZ (virus-drug complex).

### Molecular modeling

PyMOL was used for model building of MADAL385 fragments, their assembly into larger fragments and the full dendrimer, as well as for trajectory visualization and 3D figure generation [45]. Geometry optimization and point charge derivation for suitably capped fragments, namely, the pentaerythritol core, the trivalent spacer, and the peripheral N-acetylated Trp residues, were achieved by means of the PM3 hamiltonian available in the sqm program [46]. The standard ff14SB force field parameter set in AMBER 16 was used for both ligand and protein atoms [47,48]. A three-dimensional cubic grid consisting of 65x65x65 points with a spacing of 0.375 Å centered on the VP1 pore region displaying electron density for MADAL was defined for docking purposes. Electrostatic, desolvation, and affinity maps for the atom types present in MADAL385 were calculated using AutoGrid 4.2.6 and thereafter the Lamarckian genetic algorithm implemented in AutoDock4 was used to generate 100 docked conformations of a large variety of incrementally larger fragments [49]. Intra- and intermolecular energy evaluation of each configuration allowed the selection of the 10 best scoring solutions for each fragment. Significant clustering of solutions with the best scores was apparent for the smaller fragments and visual inspection confirmed the feasibility of the best binding poses, which were used for model building of the VP1 complex with the full dendrimer. For conformational sampling of the full dendrimer, MADAL385 was immersed in a cubic solvent box (TIP3P water molecules) extending 12 Å away from any ligand atom and neutralized by addition of 12 sodium ions to the locations with the most negative molecular electrostatic potential. The same procedure was later used for the VP1:MADAL385 complex; in this case, a weak harmonic restraint of 5 kcal mol^-1^ Å^-2^ on the protein Cα atoms was used throughout. Thereafter, all hydrogens and water molecules in both systems were first reoriented in the electric field of the solute and then all atoms were relaxed by performing 25 000 steps of steepest descent followed by 100 000 steps of conjugate gradient energy minimization. The resulting geometry-optimized coordinate sets were used as input for the ensuing molecular dynamics (MD) simulations at 300 K and 1 atm using the *pmemd*.*cuda* engine, as implemented in AMBER 16 [50]. The application of SHAKE to all bonds allowed an integration time step of 2 fs to be used. A cutoff distance of 9 Å was selected for the nonbonded interactions and the list of nonbonded pairs was updated every 25 steps. Periodic boundary conditions were applied and electrostatic interactions were represented using the smooth particle mesh Ewald method with a grid spacing of 1 Å [51]. The coupling constants for the temperature and pressure baths were 1.0 and 0.2 ps, respectively. Water molecules and counterions were first equilibrated around the positionally restrained solute for a first run of 0.5 ns. For the remaining 150 ns of simulation the whole systems were allowed to relax and coordinates were saved every 0.1 ns for further analysis by means of the *cpptraj* module in AMBER [52]. Subsequently, a simulated annealing procedure was followed to cool down snapshots taken every 5 ns from 300 to 273 K over a 1-ns period [53]. The geometries of these “frozen” systems were then optimized by following an energy minimization protocol until the root-mean-square of the Cartesian elements of the gradient was less than 0.01 kcal·mol-1·Å 1. The final ensemble containing 30 energy-minimized frozen molecules, which can be expected to be closer to the global energy minimum, were taken as representative of the dendrimer and the VP1-MADAL385 complex.

## Supporting information

S1 Fig **(A)** Chemical structure of suramin and pirodavir. **(B)** Comparison of the *in vitro* antiviral efficacy of MADAL385, suramin and pirodavir in a CPE-based assay. Error bars represent the mean ± SD of at least 2 independent experiments with 3 replicates.
(TIF)

S2 Fig **(A)** Replication kinetics of EV-A71 BrCr, VP1_S184T, VP1_P246S and VP1_S184T_P246S. EV-A71 Infections were determined by end-point titration at indicated time-points post infection. **(B)** Sequence alignment of the VP1 of EV-A71 clinical isolates and the lab-adapted strain BrCr. The amino acid (AA) positions in BrCr which are different from clinical isolates are highlighted in red, and clinical isolates are highlighted in blue. The compound resistant AA substitutions are highlighted in grey. **(C)** Combination study of MADAL385 and suramin with Mac synergy method. Mean volumes of synergy are presented based on 99.9% confidence values using the MacSynergy II template (Prichard and Shipman). Values above, under and in the zero plane indicate synergistic, antagonistic and additive activity, respectively. Error bars represent the mean ± SD of at least 2 independent experiments with three replicates.
(TIF)

S3 Fig Cryo-EM and 2D class average images. **(A)** A representative micrograph (out of 2,431 micrographs) (top) and selected 2D class averages (bottom) of EV-A71_11316 strain in vitreous ice. **(B)** A representative micrograph (out of 2,264 micrographs) (top) and selected 2D class averages (bottom) of EV-A71_11316 incubated with MADAL385.
(TIF)

S4 Fig Comparison of the pocket factor before and after drug incubation. **(A)** A superimposition of the atomic models without drug incubated (green) and with drug incubated (magenta) showing the pocket factor and neighboring residues. **(B and C)** Cryo-EM densities for the pocket factor and pocket area. Slab densities from the EV-A17_11316 map (B) and virus-MADAL385 complex map (C) were shown in grey mesh in addition to the corresponding atomic models. Densities connecting the pocket factor with VP1 residues that are weaker in the complex map were marked by asterisks in (C). The blue star marks the site of VP3 Ile-23. Although this site has a connecting density without drug (B), there is no pocket factor-connecting density when the drug is present.
(TIF)

S5 Fig **(A)** Superposition of 10 cooled and energy-minimized structures from the MD simulation, each separated by 5 ns and covering a total simulation time of 50 ns. Each VP1 subunit is displayed in a different color and MADAL385 is shown as sticks, with C-atoms colored in grey. **(B)** Simplified cartoon representation of VP1 in complex with MADAL385 (sticks). The view is rotated 90 degrees about the X-axis with respect to that shown in (A). **(C)** Detail from one of the complexes shown in (B) in which each VP1 subunit displays as sticks the side chains of MADAL385-interacting residues K244, T245, and P246.
(TIF)

S6 Fig Solvent-corrected binding energies (kcal·mol−1) between MADAL385 and individual residues in EV-A71 VP1. The values shown represent the averages ± SD of 30 complexes collected from the MD simulation (one every 5 ns), cooled down over 1 ns and energy minimized for geometry optimization (see Methods for details). For simplicity of representation, a cutoff of -3 kcal mol^−1^ is used.
(TIF)

S7 Fig The PSGL1-Fc fusion protein was over-expressed in HEK-293 cells and the correct size of the expression protein was verified by Western-blot using an anti-human IgG Fc antibody. (TIF)

S8 Fig Binding assay of EV-A71 BrCr in presence of cyclosporine A (4μM), a known inhibitor of cyclophilin A. The bound virus was calculated by RT-qPCR and the graph shows the % of binding relative to the untreated group. Error bars represent the mean ± SD of two independent experiments with four replicates, each.
(TIF)

S1 Movie Animation showing a representative stretch of the 150-ns trajectory of the molecular dynamics simulation (45–50 ns) of the VP1-MADAL385 complex (1024 protein residues) using the AMBERff14SB force field. For the sake of simplicity, hydrogens, sodium ions (2) and water molecules (55,359) have been omitted. Individual snapshots were created with PyMOL 1.8 (https://pymol.org/) and the frames were converted into an animated Graphics Interchange Format (gif) file by using the ImageMagick 6.7.8 suite of tools (https://www.imagemagick.org/).
(GIF)

S1 Table Cryo-EM data collection, refinement and validation statistics for the EV-A71_11316 and EV-A71_11316-MADAL385 complex structure. (DOCX)

S1 Methods (DOCX)
