# Supplementary figures and images for: Viral engagement with host receptors blocked by a novel class of tryptophan dendrimers that targets the 5-fold-axis of the enterovirus-A71 capsid
Source: PLoS Pathog. 2019 May 9;15(5):e1007760. doi: 10.1371/journal.ppat.1007760 (PMC6590834; doi:10.1371/journal.ppat.1007760)

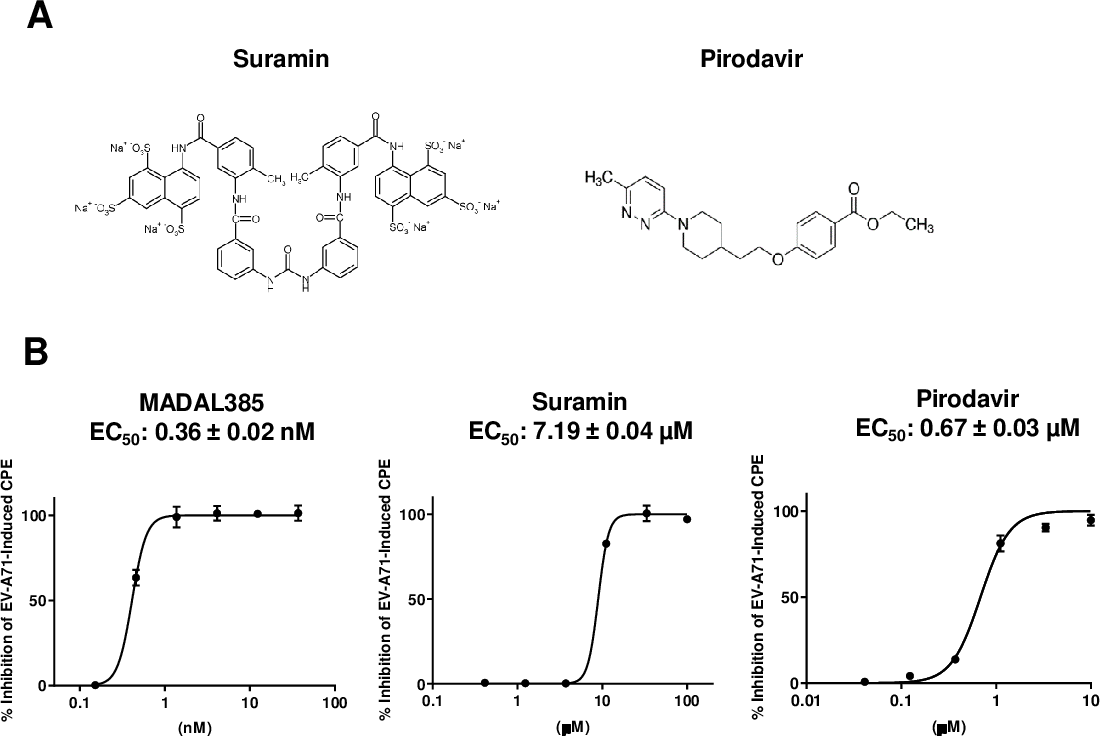

Supplement: S1 Fig — (A) Chemical structure of suramin and pirodavir. (B) Comparison of the in vitro antiviral efficacy of MADAL385, suramin and pirodavir in a CPE-based assay. Error bars represent the mean ± SD of at least 2 independent experiments with 3 replicates. (TIF) [file ppat.1007760.s001.tif]

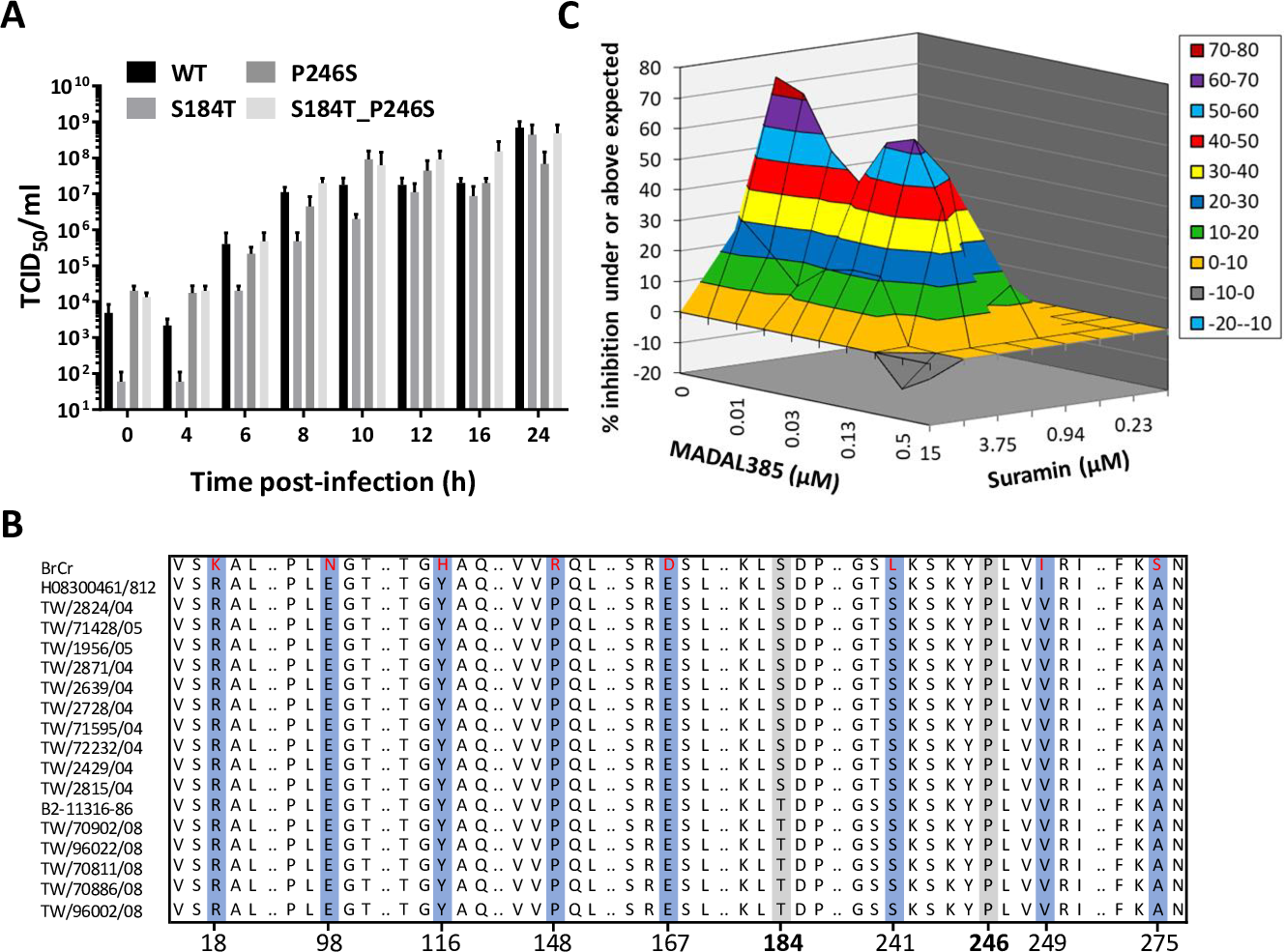

Supplement: S2 Fig — (A) Replication kinetics of EV-A71 BrCr, VP1_S184T, VP1_P246S and VP1_S184T_P246S. EV-A71 Infections were determined by end-point titration at indicated time-points post infection. (B) Sequence alignment of the VP1 of EV-A71 clinical isolates and the lab-adapted strain BrCr. The amino acid (AA) positions in BrCr which are different from clinical isolates are highlighted in red, and clinical isolates are highlighted in blue. The compound resistant AA substitutions are highlighted in grey. (C) Combination study of MADAL385 and suramin with Mac synergy method. Mean volumes of synergy are presented based on 99.9% confidence values using the MacSynergy II template (Prichard and Shipman). Values above, under and in the zero plane indicate synergistic, antagonistic and additive activity, respectively. Error bars represent the mean ± SD of at least 2 independent experiments with three replicates. (TIF) [file ppat.1007760.s002.tif]

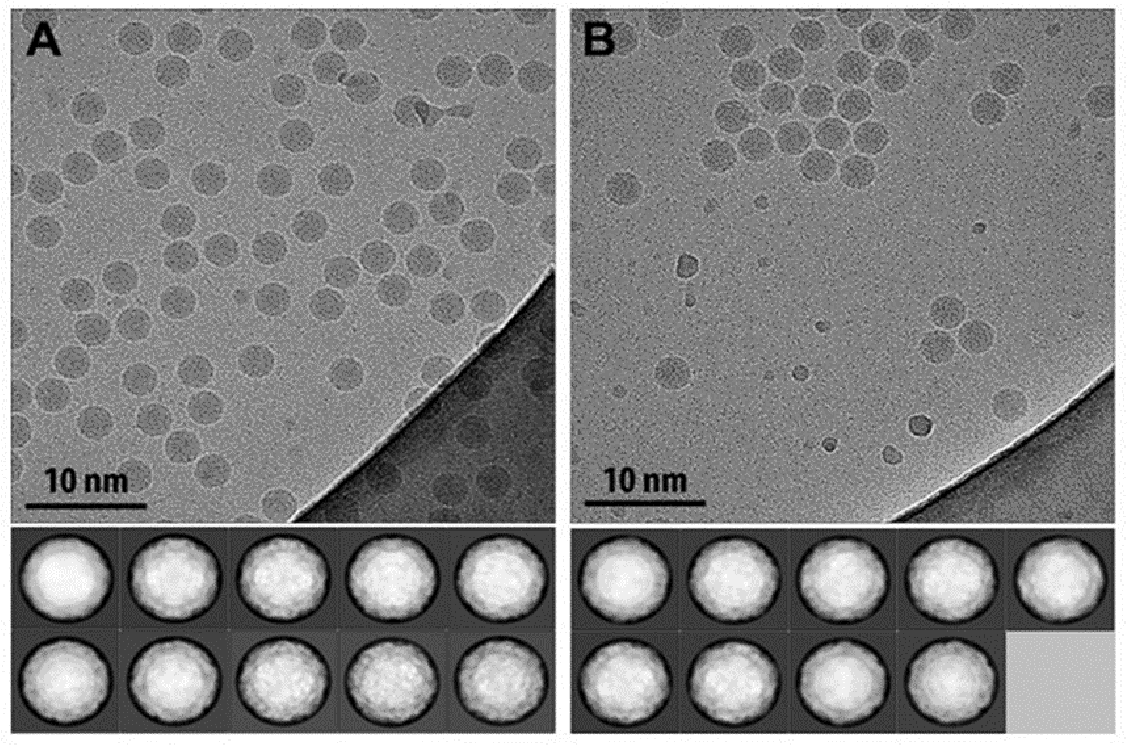

Supplement: S3 Fig — (A) A representative micrograph (out of 2,431 micrographs) (top) and selected 2D class averages (bottom) of EV-A71_11316 strain in vitreous ice. (B) A representative micrograph (out of 2,264 micrographs) (top) and selected 2D class averages (bottom) of EV-A71_11316 incubated with MADAL385. (TIF) [file ppat.1007760.s003.tif]

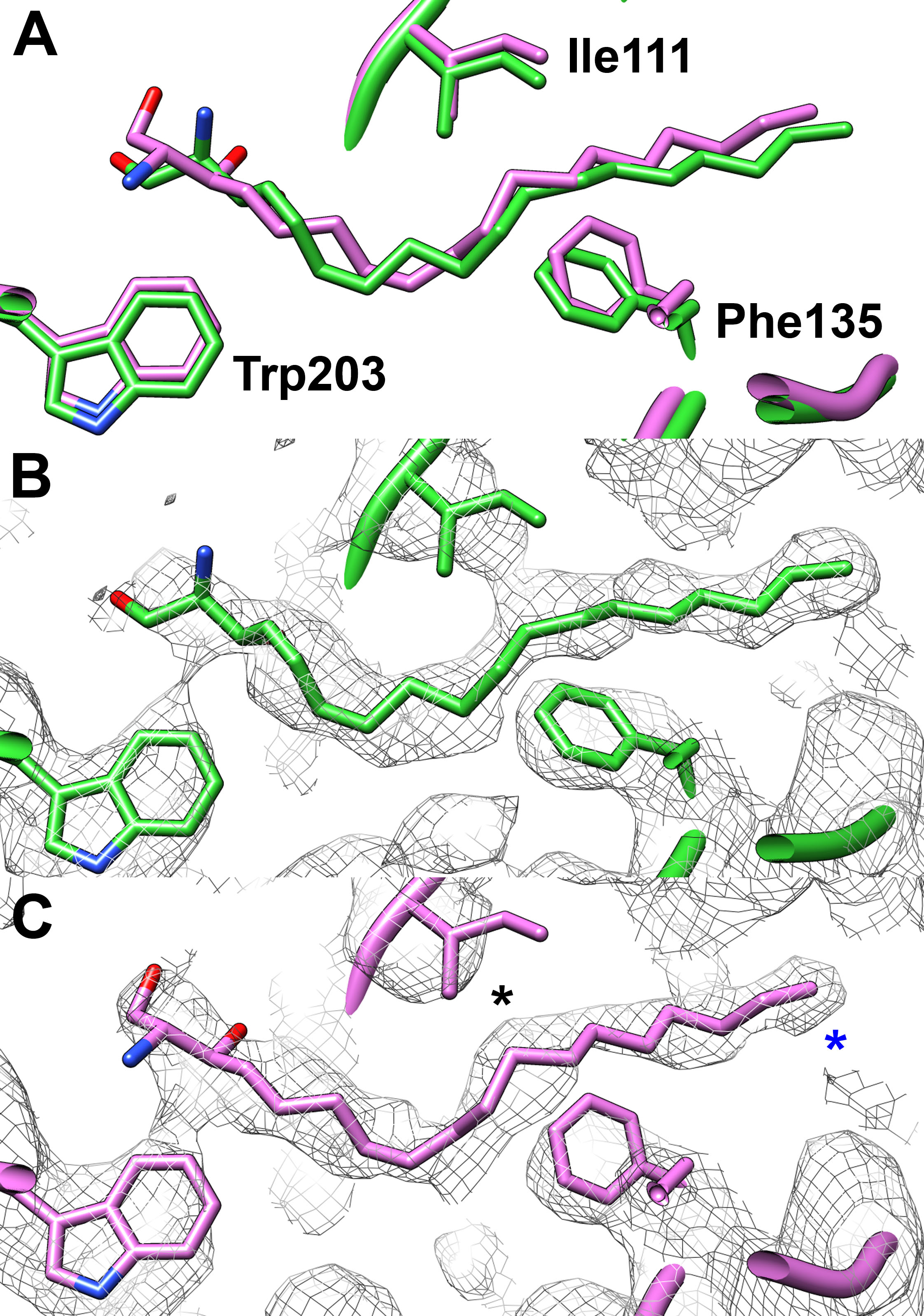

Supplement: S4 Fig — (A) A superimposition of the atomic models without drug incubated (green) and with drug incubated (magenta) showing the pocket factor and neighboring residues. (B and C) Cryo-EM densities for the pocket factor and pocket area. Slab densities from the EV-A17_11316 map (B) and virus-MADAL385 complex map (C) were shown in grey mesh in addition to the corresponding atomic models. Densities connecting the pocket factor with VP1 residues that are weaker in the complex map were marked by asterisks in (C). The blue star marks the site of VP3 Ile-23. Although this site has a connecting density without drug (B), there is no pocket factor-connecting density when the drug is present. (TIF) [file ppat.1007760.s004.tif]

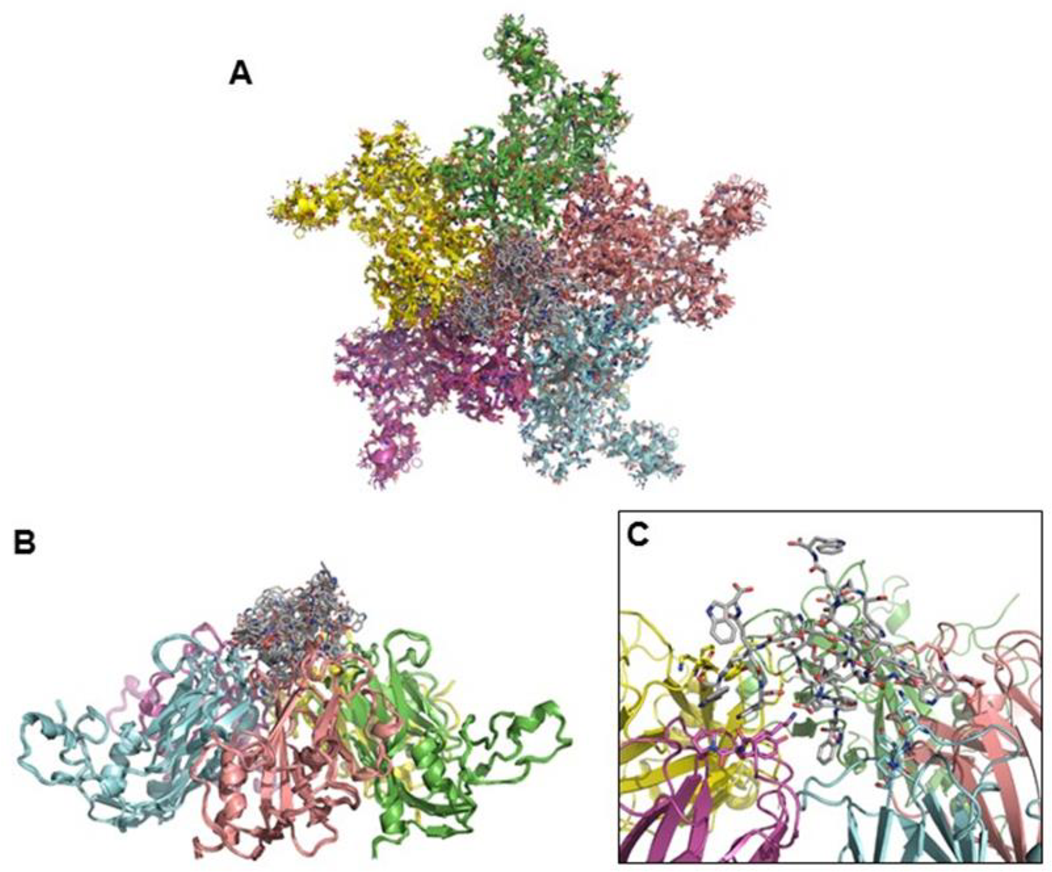

Supplement: S5 Fig — (A) Superposition of 10 cooled and energy-minimized structures from the MD simulation, each separated by 5 ns and covering a total simulation time of 50 ns. Each VP1 subunit is displayed in a different color and MADAL385 is shown as sticks, with C-atoms colored in grey. (B) Simplified cartoon representation of VP1 in complex with MADAL385 (sticks). The view is rotated 90 degrees about the X-axis with respect to that shown in (A). (C) Detail from one of the complexes shown in (B) in which each VP1 subunit displays as sticks the side chains of MADAL385-interacting residues K244, T245, and P246. (TIF) [file ppat.1007760.s005.tif]

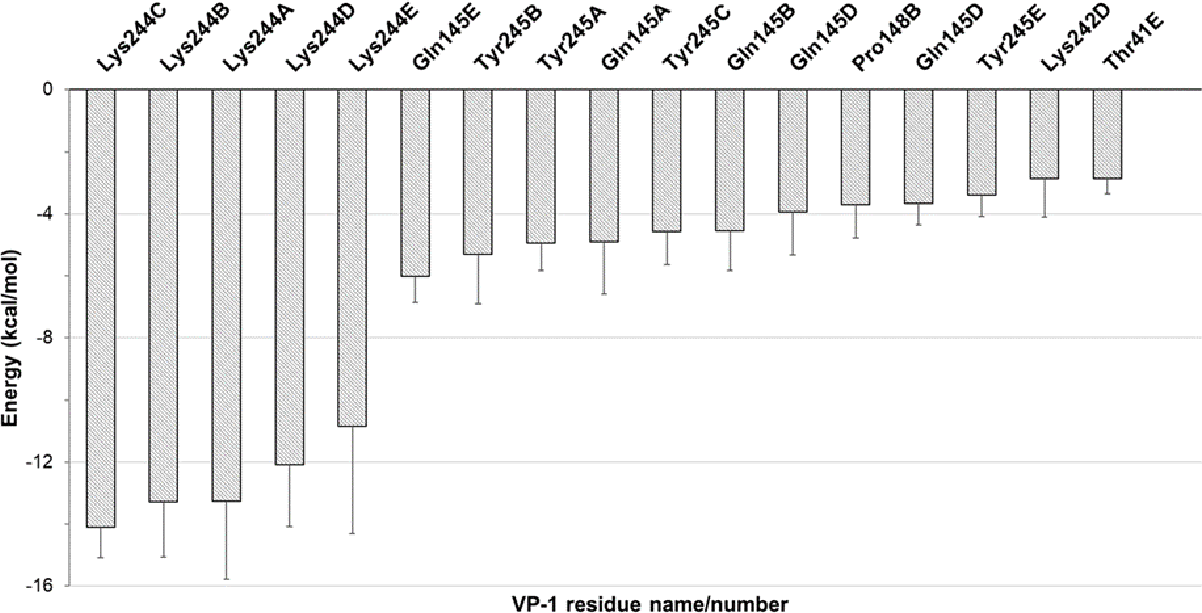

Supplement: S6 Fig — The values shown represent the averages ± SD of 30 complexes collected from the MD simulation (one every 5 ns), cooled down over 1 ns and energy minimized for geometry optimization (see Methods for details). For simplicity of representation, a cutoff of -3 kcal mol−1 is used. (TIF) [file ppat.1007760.s006.tif]

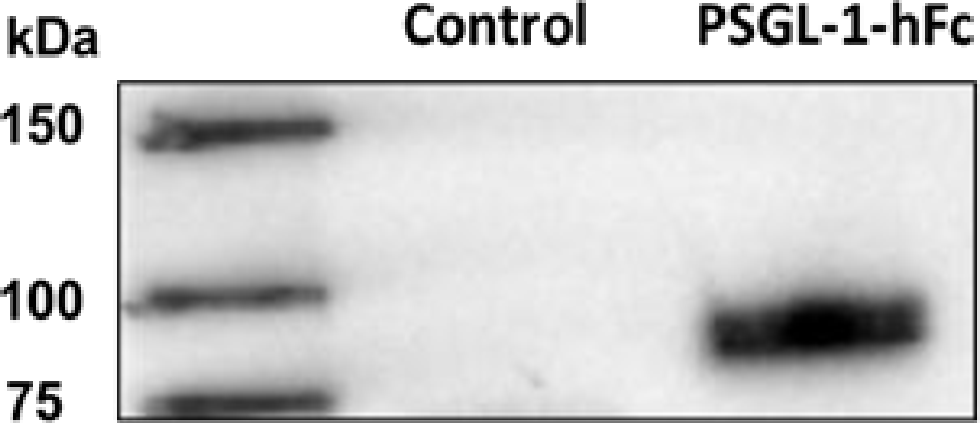

Supplement: S7 Fig — (TIF) [file ppat.1007760.s007.tif]

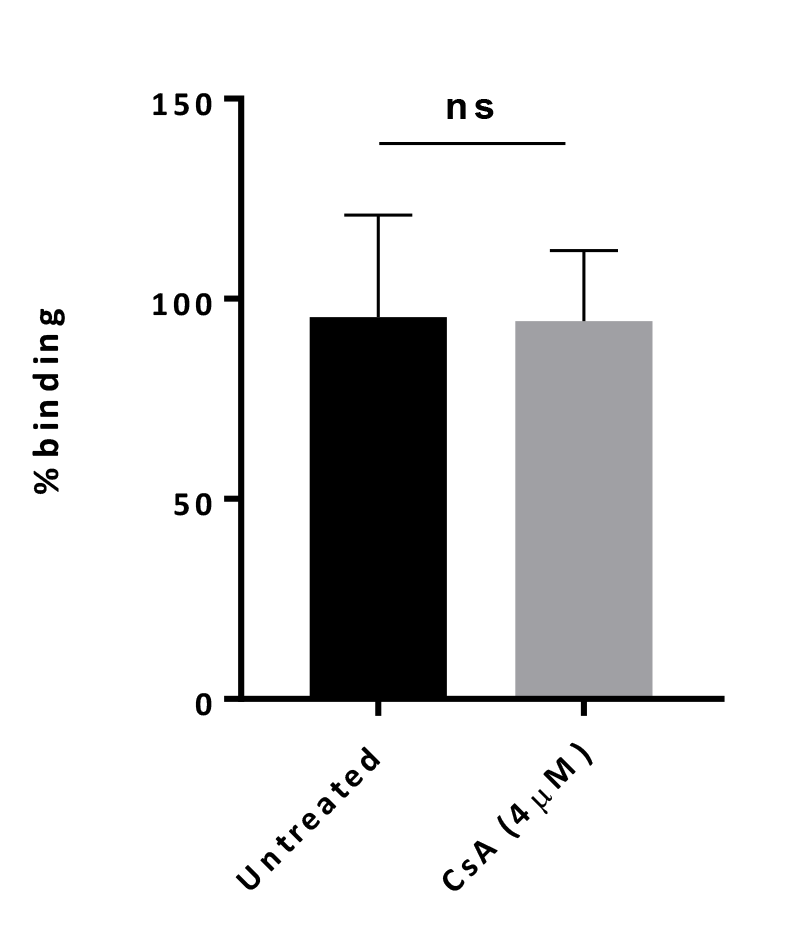

Supplement: S8 Fig — The bound virus was calculated by RT-qPCR and the graph shows the % of binding relative to the untreated group. Error bars represent the mean ± SD of two independent experiments with four replicates, each. (TIF) [file ppat.1007760.s008.tif]

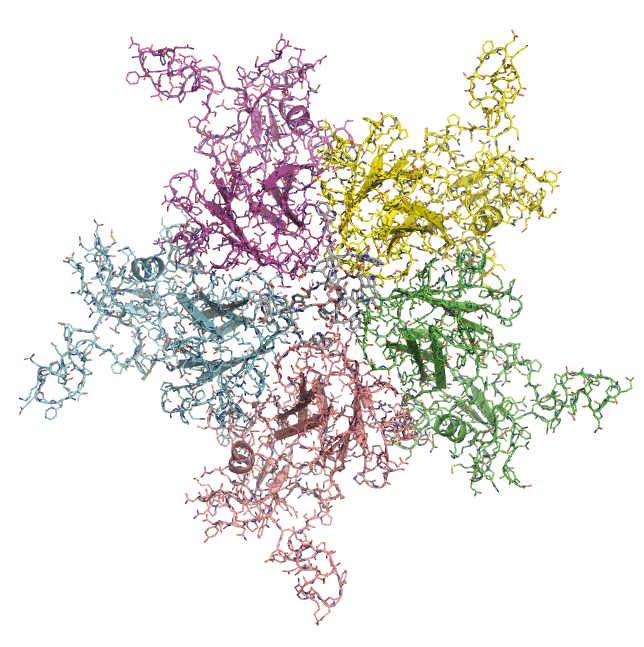

Supplement: S1 Movie — For the sake of simplicity, hydrogens, sodium ions (2) and water molecules (55,359) have been omitted. Individual snapshots were created with PyMOL 1.8 (https://pymol.org/) and the frames were converted into an animated Graphics Interchange Format (gif) file by using the ImageMagick 6.7.8 suite of tools (https://www.imagemagick.org/). (GIF) [file ppat.1007760.s009.gif]
