# Supplementary material for: Viral engagement with host receptors blocked by a novel class of tryptophan dendrimers that targets the 5-fold-axis of the enterovirus-A71 capsid
Source: PLoS Pathog. 2019 May 9;15(5):e1007760. doi: 10.1371/journal.ppat.1007760 (PMC6590834; doi:10.1371/journal.ppat.1007760)
Supplement: S1 Table — (DOCX) [file ppat.1007760.s010.docx]

**S1 Table.** Cryo-EM data collection, refinement and validation statistics for the EV-A71_11316 and EV-A71_11316-MADAL385 complex structure.

|  | EV-A71  (EMDB-7905)  (PDB 6DIJ) | EV-A71 + MADAL385  (EMDB-7913)  (PDB 6DIZ) |
| --- | --- | --- |
| **Data collection and processing** |  |  |
| Magnification | 59,000x | 59,000x |
| Voltage (kV) | 300 | 300 |
| Electron exposure (e^–^/Å^2^) | 45 | 46 |
| Defocus range (μm) | 0.5 – 4.3 | 0.6 – 4.4 |
| Pixel size (Å) | 1.1 | 1.1 |
| Micrographs (no.) | 2,431 | 2,264 |
| Symmetry imposed | Icosahedral | Icosahedral |
| Particles images (no.) | 152,476 | 11,813 |
| Map resolution (Å) | 3.3 | 3.6 |
| FSC threshold | 0.143 | 0.143 |
|  |  |  |
| **Refinement** |  |  |
| Initial model used (PDB code) | 3VBS | 3VBS |
| Map sharpening B-factor (Å^2^) | -256 | -219 |
| Model composition |  |  |
| Non-hydrogen atoms | 6,536 | 6,536 |
| Protein residues | 842 | 842 |
| Ligand atoms | 21 | 21 |
| R.m.s. Deviations |  |  |
| Bond lengths (Å) | 0.009 | 0.007 |
| Bond Angles (°) | 0.943 | 0.826 |
| Validation |  |  |
| Molprobity score | 1.40 | 1.53 |
| Clashscore | 1.47 | 2.87 |
| Poor rotamers (%) | 0.42 | 0.28 |
| Ramachandran plot |  |  |
| Favored (%) | 91.0 | 92.8 |
| Allowed (%) | 100.0 | 100.0 |
| Disallowed (%) | 0.0 | 0.0 |
