# Supplementary material for: Viral engagement with host receptors blocked by a novel class of tryptophan dendrimers that targets the 5-fold-axis of the enterovirus-A71 capsid
Source: PLoS Pathog. 2019 May 9;15(5):e1007760. doi: 10.1371/journal.ppat.1007760 (PMC6590834; doi:10.1371/journal.ppat.1007760)
Supplement: S1 Methods — (DOCX) [file ppat.1007760.s011.docx]

**S1 Methods**

**Binding and internalization inhibition assay**

EV-A71 viruses were incubated with the compound at 37°C for 1h, after which the mixture was used to infect RD cells at 4°C for 1h with EV-A71 BrCr or 812 strain (MOI= 3). Followed by extensive washes, detection of cell-bound viral particles was performed by RT-qPCR. After attachment of EV-A71 viruses to RD cells at 4°C for 1h, RD cells were incubated at 37°C for 1h to allow virus internalization in the presence/absence of compound. Then, infected RD cells were treated with trypsin to remove bound-virus from the cell surface. Detection of intracellular virus was performed by RT-qPCR.

**Thermostability assay**

EV-A71 BrCr was incubated in the presence of MADAL (15 µM), pirodavir (15 µM), suramin (150 µM) and equal final concentration of DMSO for 15 min at 37° C. Following the incubation for 2 min at different temperature ranging from 37° C to 52° C, samples were cooled rapidly to 4°C. Subsequently, the infectious viral load was determined in each condition by end-point titration on RD cells.

**Neutralization assay**

Increasing concentrations of heparin (Heparin sodium salt from porcine intestinal mucosa, Sigma-Aldrich) were incubated with EV-A71 BrCr (WT), EV-A71 resistant strain (VP1_S184P_P246S) or EV-A71 sensitive strain [VP1(11316)] for 1h at 37°C. Next, RD cells were infected with these mixtures for 1h at 37°C. After removing the inoculum and washing, cells were incubated at 37°C for 3 days. Heparin EC_50_ was then calculated as the concentration of Heparin responsible for neutralization of 50% of viral infection.

**Generation of tryptophan dendrimer-resistant EV-A71 viruses**

The clonal selection of EV-A71 variants resistant to tryptophan dendrimer was achieved with a five-step protocol. Briefly, MOI-dependent assay was performed on RD cells to determine the combination of compound (the lowest concentration 2.8 µM) and virus (the highest EV-A71 input, MOI = 1) where complete CPE-inhibition was still observed. Subsequently, this combination was applied to two 96-well plates of RD cells and after 3-4 days incubation, the supernatants from the wells, in which virus induced-CPE was observed, were harvested. To purify and enrich the putative resistant variants, the harvested supernatants were titrated in the presence of the same concentration of tryptophan dendrimer. The highest dilution of compound-resistant virus was then collected and inoculated in cell culture flasks (in presence of compound) to produce resistant variants stock for further study. Genotyping was determined by Sanger sequencing of the whole genome. Finally, resistance was confirmed in cell based CPE-reduction antiviral assay in parallel with the wild-type virus.

**Reverse-engineering to generate resistant or sensitive EV-A71 variants.**

The putative compound-resistant or compound-sensitive variants were introduced into the EV-A71 BrCr infectious clone using QuickChange Site-Directed Mutagenesis Kit (Agilent Genomics) according to the manufacturer’s instructions. After verification by sequencing, the linearized template DNA (Mlu 1, Promega) was *in vitro* retro-transcribed by using T7 RiboMAX^TM^ Express Large Scale RNA Production System (Promega). The transcribed RNA was transfected into RD cell monolayers in 6-well plate with TransIT-mRNA Transfection Kit (Mirus Bio LLC). CPE was typically observed at 48-72h post transfection and supernatants were harvested and stored at -80°C. To confirm the mutation, viral RNA was extracted using NucleoSpin RNA kit, and full genome sequencing was performed after RT-PCR.

**Image processing**

Both data sets (EV-A71_11316 and EV-A71_11316-MADAL385 complex) were processed in the same manner. Micrograph movie frame stacks were aligned and dose-weighted with UCSF program MotionCorr v2.1 [1] and the contrast transfer function (CTF) of each micrograph was determined with GCTF [2]. Image processing steps were performed by using RELION v2.1 [3]. After an initial particle picking for a subset of the data, particles were extracted with a 340-pixel by 340-pixel box and processed for reference-free 2D classification. Using the output 2D classes as references, 174,637 and 27,450 particles were picked by automatic particle picking for virus-alone and the complex data sets, respectively [4]. The extracted particles were subjected to 2D- and 3D-classfiication, yielding 152,476 and 11,813 particles. An *ab initio* 3D model was generated with icosahedral-symmetry averaging by a stochastic gradient descent algorithm in RELION, followed by 3D refinement. 3.3 Å and 3.6 Å resolution maps for the virus-alone and the complex (FSC = 0.143) were reconstructed after post-processing.

**Atomic coordinate refinement**

A homology model of EV-A71_11316 was generated from the EV71 crystal structure (PDB ID 3VBS) by using swiss-model [5]. Building of the model into the sharpened map was initiated by fitting the homology model as a rigid body in Chimera [6]. The fitted structure of an asymmetric unit was duplicated for sixty icosahedral units and refined with non-crystallographic symmetry using PHENIX real-space refinement [7]. The map cross-correlation values for ‘whole unit cell’ and ‘around atoms’ were 0.680 and 0.874, respectively. Root-mean-square deviation (RMSD) deviations from ideal bonds length and angles were 0.009 Å and 0.943º for the virus 0.007 Å and 0.826º for the complex. Ramachandran outliers were 0.00%. The refined model was visually inspected, modified in COOT [8], and validated by Molprobity [9].**References**

1. Zheng SQ, Palovcak E, Armache J-P, Verba KA, Cheng Y, Agard DA. MotionCor2: anisotropic correction of beam-induced motion for improved cryo-electron microscopy. Nat Methods. 2017;14: 331–332. doi:10.1038/nmeth.4193

2. Zhang K. Gctf: Real-time CTF determination and correction. J Struct Biol. 2016;193: 1–12. doi:10.1016/j.jsb.2015.11.003

3. Kimanius D, Forsberg BO, Scheres S, Lindahl E. Accelerated cryo-EM structure determination with parallelisation using GPUs in RELION-2. 2016 Jun. Report No.: biorxiv;059717v1.

4. Scheres SHW. Semi-automated selection of cryo-EM particles in RELION-1.3. J Struct Biol. 2015;189: 114–122. doi:10.1016/j.jsb.2014.11.010

5. Biasini M, Bienert S, Waterhouse A, Arnold K, Studer G, Schmidt T, et al. SWISS-MODEL: modelling protein tertiary and quaternary structure using evolutionary information. Nucleic Acids Res. 2014;42: W252–W258. doi:10.1093/nar/gku340

6. Pettersen EF, Goddard TD, Huang CC, Couch GS, Greenblatt DM, Meng EC, et al. UCSF Chimera—A visualization system for exploratory research and analysis. J Comput Chem. 2004;25: 1605–1612. doi:10.1002/jcc.20084

7. Afonine P V., Headd JJ, Terwilliger TC, Adams PD. PHENIX News. Comput Crystallogr Newsl. 2013;4: 43–44.

8. Emsley P, Lohkamp B, Scott WG, Cowtan K. Features and development of Coot. Acta Crystallogr Sect D Biol Crystallogr. 2010;66: 486–501.

9. Chen VB, Arendall WB, Headd JJ, Keedy DA, Immormino RM, Kapral GJ, et al. *MolProbity* : all-atom structure validation for macromolecular crystallography. Acta Crystallogr Sect D Biol Crystallogr. 2010;66: 12–21. doi:10.1107/S0907444909042073
